# Bio-Inspired Cross-Spiral Carbon Fiber Composites: Impact Resistance and Damage Tolerance Under Multiple Low-Velocity Impact

**DOI:** 10.3390/polym18101162

**Published:** 2026-05-09

**Authors:** Lanlan Jiang, Dongfeng Li, Zaoyang Guo

**Affiliations:** School of Science, Harbin Institute of Technology, Shenzhen 518055, China; jiang_llan@163.com

**Keywords:** fiber-reinforced polymer composite structures, multiple low-velocity impact, impact resistance, CAI strength

## Abstract

This study investigates the impact resistance and damage tolerance of bio-inspired cross-spiral (CS) laminates under multiple low-velocity impacts. Two impact conditions were considered: repeated impacts at the same location and double impacts at different locations. Low-velocity impact tests, ultrasonic C-scan inspection, and compression-after-impact (CAI) tests were conducted to evaluate the impact response, internal damage, and residual compressive strength. The results show that repeated impacts at the same location intensified deformation and damage accumulation. When the number of impacts increased from 1 to 15, the peak force and maximum central displacement increased by 20.26% and 26.21%, respectively, while the absorbed energy decreased by 70.96% and the damage area increased by 113.67%. The variations in response parameters and damage area became less pronounced as the number of impacts increased. Under double impacts at different locations, increasing the impact spacing reduced damage coupling between the two impacts. When the spacing increased from 0 mm to 80 mm, the CAI strength increased by 11.02%. Overall, repeated impacts at the same location reduced the residual compressive load-bearing capacity, whereas larger impact spacing helped improve the CAI performance.

## 1. Introduction

Fiber-reinforced polymer composite structures may be subjected to complex impact loading conditions during service, such as foreign object impacts from runway debris or tire fragments on fuselage, wing and fairing components during aircraft takeoff or landing, and low-velocity collisions between hull structures and floating objects in marine environments. These impacts may cause fiber fracture, interfacial debonding and matrix cracking [1,2,3,4]. During routine maintenance and service, composite structures are often exposed to even more complex multiple impact environments, where the resulting damage accumulation tends to exacerbate structural failure and degradation [5,6,7]. Biomimetics draws on structural systems refined by long-term natural selection and translates specific features found in nature into engineering design [8]. Based on different biological structural characteristics, multi-level and multi-scale biomimetic design and material innovation have been explored. Among the diverse structural configurations found in nature, helicoidal layered structures have attracted increasing attention because of their distinctive structural characteristics.

**Figure 1:**
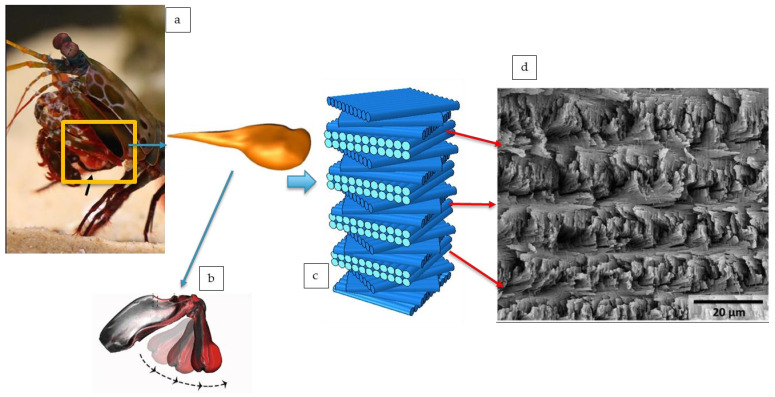
Schematic illustration of the mantis shrimp dactyl club, the natural spiral structure, and the corresponding SEM morphology: (**a**) Mantis shrimp; (**b**) Dactyl club; (**c**) Schematic of the helicoidal structure; (**d**) SEM image of the dactyl club fracture surface [10].

Spiral structures represent a common hierarchical configuration in natural layered materials, in which fibers or fiber bundles are progressively rotated and stacked at specific angular intervals along the thickness direction [9]. As shown in Figure 1 [10], the spiral structure in the dactyl club of the peacock mantis shrimp consists of multiple rotated fibrous units [11,12]. Similar structural features have also been reported in biological materials such as coelacanth scales [13,14,15]. In fish scales and bone tissues, these fibers are mainly composed of mineralized collagen, whereas in arthropod exoskeletons, chitin fibers are commonly embedded in a relatively soft protein matrix to form a composite system [16]. The peacock mantis shrimp uses its dactyl club for predation, defense, and intraspecific competition. Although the dactyl club is small, a single strike can be completed within milliseconds, with a peak velocity exceeding 20 m/s, an acceleration of up to 10^5^ m/s^2^, and an instantaneous impact force of up to 1.5 kN [17,18]. Such high velocity and high load acting on a small structure can generate substantial local stress. Nevertheless, the dactyl club can withstand thousands of repeated impacts without obvious failure, indicating the excellent impact damage resistance associated with its spiral structure. In the mantis shrimp dactyl club, the spiral angle between adjacent chitin fiber layers is approximately 1.6–6.2°, and each structural unit completes a 180° rotation [19]. This spiral arrangement can alter the propagation paths of cracks and delaminations, while debonding, sliding, and friction at the fiber–matrix interface contribute to energy dissipation.

Experimental studies on the low-velocity impact behavior of bio-inspired spiral composite laminates have mainly focused on single-impact conditions, whereas research on multiple impacts remains relatively limited. Existing work has generally compared the impact resistance and damage tolerance of spiral and conventional layups in terms of peak force, absorbed energy, and compression-after-impact (CAI) strength. Inspired by the spiral structure in crustacean exoskeletons, Feng et al. [20] designed linear spiral composite laminates and found that, under their test conditions, these laminates exhibited higher impact resistance and greater energy absorption capacity than conventional cross-ply laminates. Liu et al. [21] reported that, in their comparison, spiral configurations showed higher impact resistance than conventional configurations because the spiral arrangement altered the crack propagation path and delayed the formation and growth of through-thickness cracks. Ginzburg et al. [22] demonstrated in their study that spiral structures exhibited improved damage tolerance under higher impact energies, and further noted that their ability to disperse impact energy along the laminate plane became more pronounced as the in-plane dimensions increased. The results reported by Liu et al. [23], Wang et al. [24], Lim et al. [25], and Zhang et al. [26] further indicated that spiral layups can improve laminate toughness, penetration resistance, and peak force, although damage tends to propagate along the laminate plane in the form of delamination and matrix cracking, while concentrated through-thickness failure is suppressed. In spiral structure design, the helix angle, spiral configuration, and related geometric parameters are important factors governing the impact response. Chew et al. [27], Guan et al. [28], Lu et al. [29], and Du et al. [30] showed that the helix angle affects the out-of-plane load-bearing capacity, absorbed impact energy, impact force, and damage area, although the specific trends depend on the material system. The design of spiral structures has also gradually evolved from single-spiral configurations to coupled multi-spiral forms. Yin et al. [31] compared single- and double-spiral composite structures with helix angles of 10° and 20° through pendulum impact tests and found that, under their test conditions, the double-spiral configurations absorbed more energy than the corresponding single-spiral configurations. Related findings on double-spiral configurations were also presented by Rai et al. [32] and Yan et al. [33]. Ouyang et al. [34] showed in their comparative study that double-spiral laminates exhibited relatively high impact peak forces but were more prone to internal damage propagation, whereas cross-spiral configurations showed higher peak forces and smaller damage areas under different conditions. Han et al. [35] extended the comparison to linear, nonlinear, and combined spiral configurations and found that, in their tests, the combined configuration exhibited higher peak force and bending stiffness under 40 J and 80 J impacts. In terms of CAI performance, Zhang et al. [26] observed in their study that spiral layups could withstand higher peak forces during impact and retain higher residual load-bearing capacity during CAI testing. Körbelin et al. [36] reduced the helix angle to 2.07° and found that, under their experimental conditions, the CAI strength of the spiral laminate remained higher than that of the 45° quasi-isotropic layup. Liu et al. [37] introduced variable-stiffness curvilinear fiber paths into conventional spiral layups, and their results showed that this design increased damage tolerance by 32.1% compared with the quasi-isotropic layup and produced higher CAI strength than the constant-stiffness linear spiral layup. Zhao et al. [38] incorporated a sinusoidal structure into spiral laminate design and found that, in their comparison, the sinusoidal spiral configuration showed higher residual strength than the parallel spiral configuration.

Research on multiple low-velocity impact remains limited at present, and the few existing studies have primarily focused on repeated impacts at the same location. For instance, in the work of Yang et al. [39], double-spiral laminates showed higher energy absorption capacity under single impact and also exhibited more favorable impact resistance than single-spiral configurations under repeated low-velocity impacts at a single point. Han et al. [40], based on their study of basalt fiber-reinforced bio-inspired spiral composite laminates, observed that double-spiral laminates achieved higher maximum contact forces under 8 J, 12 J, and 16 J conditions, and also presented the lowest overall damage after five consecutive impacts at the same point. Furthermore, a comparison between cross-spiral and symmetric spiral configurations revealed that as the number of impacts increases, the cross-spiral configuration consistently shows smaller central displacement and less severe damage accumulation [41].

It can be seen that current research on the low-velocity impact behavior and damage tolerance of bio-inspired spiral composite laminates is still mainly focused on single low-velocity impact conditions, while studies on multiple low-velocity impacts remain relatively limited. The few available studies on multiple impacts have mostly addressed the impact resistance under repeated impacts at the same location, with comparatively limited attention given to the damage tolerance of laminates under this condition. In addition, research on impacts at different locations is clearly insufficient, and the relationship among impact response, damage evolution, and residual mechanical properties under such conditions has not yet been systematically investigated. Therefore, inspired by naturally occurring spiral layered structures, this paper designs and fabricates bio-inspired cross-spiral laminates using a compression molding method, and investigates their multiple low-velocity impact damage behavior and damage tolerance. Under an impact energy of 10 J, two types of loading conditions are established: consecutive impacts at the same location and consecutive impacts at different locations. Through drop-weight impact tests, compression-after-impact (CAI) tests, and ultrasonic C-scanning, the dynamic response, damage accumulation characteristics, and residual properties after multiple impacts are effectively analyzed.

## 2. Materials and Methods

### 2.1. Specimen Preparation

**Figure 2:**
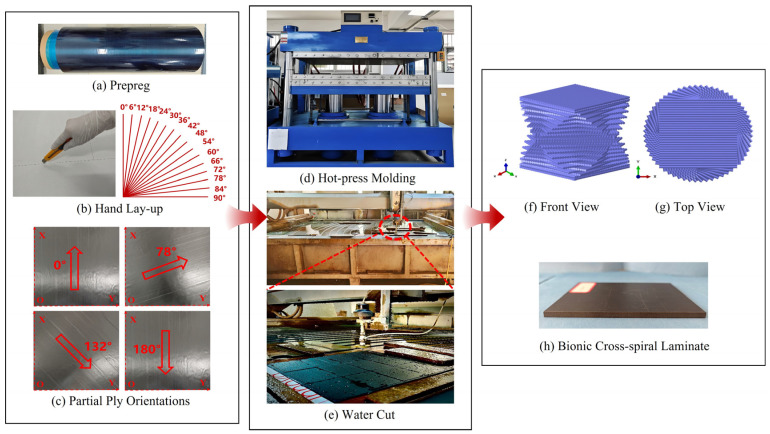
Configuration and fabrication process of bionic helicoidal composite laminates.

To investigate the impact resistance and damage tolerance of bio-inspired spiral composite laminates under multiple low-velocity impacts, a bio-inspired cross-spiral (CS) laminate was selected in this study. The laminate consisted of 32 plies with a symmetric stacking sequence of [0/90/6/84/12/78/18/72/24/66/30/60/36/54/42/48]_s_. The thickness of each ply was 0.14 mm, resulting in a total laminate thickness of 4.48 mm. The front and top views of the CS configuration are shown in Figure 2f and Figure 2g, respectively. The specimens were fabricated by manual layup using carbon fiber-reinforced prepreg supplied by Zhangjiagang Weinuo Composite Materials Co., Ltd. (Suzhou, China), as shown in Figure 2a. The prepreg consisted of T300 unidirectional carbon fibers and YPH-69 epoxy resin. The carbon fiber volume fraction was 67%, with an areal density of 150 g/m^2^, while the epoxy resin volume fraction was 33%, with a density of 1.21–1.25 g/cm^3^ and a viscosity of 30,000–40,000 cps. The prepreg was stored in roll form, with adjacent layers separated by polyethylene film and release paper to prevent adhesion, limit moisture and oxygen ingress, and avoid premature curing before molding.

To control the helix angle during manual layup, the 0° reference line on the layup table was used as the common baseline. The first ply was a 0° unidirectional prepreg, with its four edges aligned to the positioning lines on the layup table, serving as the reference ply for subsequent layup. The remaining plies were sequentially aligned with the angular scale on the layup table according to the designed ply angles. After each ply was completed, the laminate outline and angular position were checked. Layer-by-layer compaction was applied to improve interply contact and reduce ply misalignment and interply sliding. The use of the 0° reference line, edge positioning, angular-scale alignment, and layer-by-layer checking improved the reproducibility of the cross-spiral layup. Representative ply orientations are shown in Figure 2c.

**Figure 3:**
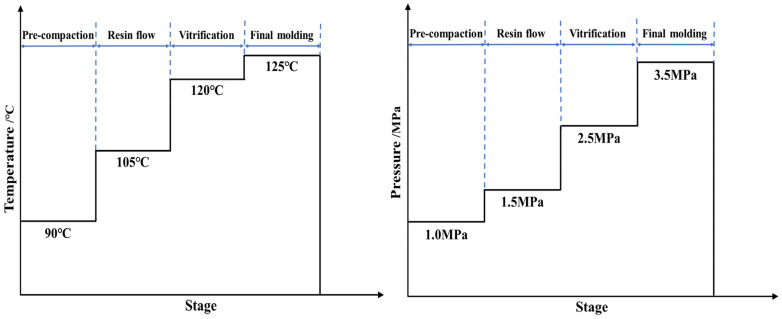
Schematic temperature and pressure profiles of the compression molding process.

After manual layup, compression molding was performed using an XL-3-17-1 hot press (Dongguan Xuli Electromechanical Equipment Co., Ltd., Dongguan, China), as shown in Figure 2d. To improve molding quality and process repeatability, the compression molding process included pre-compaction, edge trimming, film wrapping, thickness control, and molding. The corresponding temperature and pressure profiles are shown in Figure 3. Pre-pressing was performed at 90 °C and 1.0 MPa for approximately 180 s to expel entrapped air between prepreg plies, reduce void formation, and minimize the effect of initial lay-up defects. After pre-pressing, the irregular thickened regions along the laminate edges were trimmed to reduce the risk of edge porosity and warpage. Film wrapping was then applied to mitigate surface wrinkles and local indentation during compression molding, while glass fiber spacer blocks were used for thickness control.

The forming stage was divided into gelation, vitrification, and final forming. During gelation, the temperature and pressure were set to 105 °C and 1.5 MPa, respectively, and maintained for approximately 200 s. The reduced viscosity of the epoxy resin at this stage promoted matrix impregnation into the fibers and interlaminar interfaces. During vitrification, the temperature, pressure, and holding time were increased to 120 °C, 2.5 MPa, and 300 s, respectively. The resin system gradually changed from a viscous flow state to a cured state, and the laminate developed the designed geometric configuration. Final forming was conducted at 125 °C and 3.5 MPa for 3500 s, during which the epoxy resin underwent further thermal curing and the laminate geometry was fixed.

After molding, the specimens were cut according to the dimensional requirements of ASTM D7136 [42] using a YL3020 three-axis gantry waterjet cutting machine (Foshan Yuanli Precision Machinery Co., Ltd., Foshan, China). The final CS laminate specimens, shown in Figure 2h, had dimensions of 150 mm × 100 mm. The cutting pressure and cutting speed were 320 MPa and 900 mm/min, respectively.

### 2.2. Experimental Procedure

**Figure 4:**
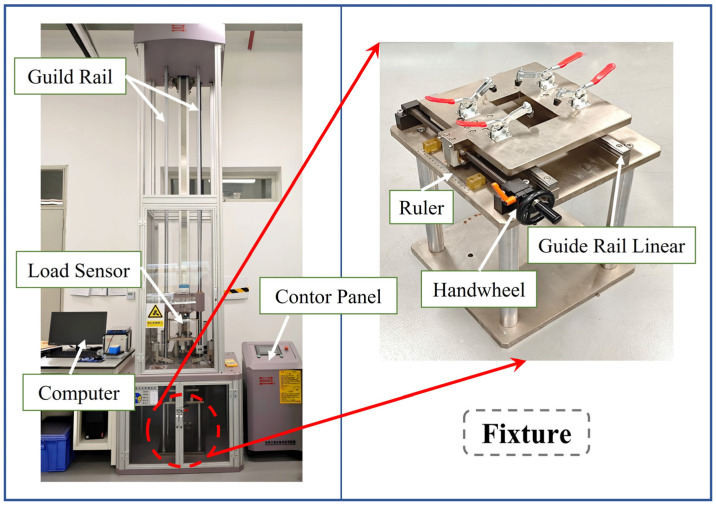
Drop-weight impact test setup.

The multiple low-velocity impact tests were conducted using a DIT 183E drop-weight impact testing machine (Shenzhen Wance Testing Equipment Co., Ltd., Shenzhen, China), as shown in Figure 4. The impactor had a mass of 5.5 ± 0.25 kg and a diameter of 16 ± 0.1 mm, with a hemispherical head. After each group of impact tests, the hemispherical tip of the impactor was inspected to confirm that there was no obvious wear, deformation, or residual material attached to the contact surface. This study considered two impact scenarios: repeated impacts at the same location and multiple impacts at two distinct locations. The impact energy was set to 10 J for each impact, corresponding to an impactor drop height of 0.185 m. All impact tests were conducted under controlled laboratory conditions, with the temperature and relative humidity maintained at 23 ± 2 °C and 50 ± 5%, respectively. During the test, impact sensors were used to acquire the load, displacement, and energy responses of the impacted specimen. To ensure the stability of the loading process, the laminate specimens were constrained on all four sides using a dedicated support fixture, as shown in Figure 4, to prevent slipping or overturning during impact. The fixture was equipped with a rigid ruler and a rotating handwheel, allowing for precise adjustment of the laminate position. The low-velocity impact tests were performed in accordance with ASTM D7136 [42], and two types of test conditions were considered: consecutive impacts at the same location and consecutive impacts at different locations. These were used to analyze the dynamic response and failure modes of the CS laminates under multiple low-velocity impacts. The impactor was aligned perpendicular to the laminate surface, and all impacts were applied along the through-thickness direction of the specimens. Oblique impact loading was not considered in this study.

This study designs experiments to investigate the dynamic mechanical response and damage tolerance of CS laminates under multiple low-velocity impacts, including two parts: consecutive impacts at the same location and multiple impacts at different locations. The low-velocity impact tests were conducted in accordance with ASTM D7136 [42]. For the consecutive impacts at the same location, the impact position was set at the geometric center of the laminate, with a single impact energy maintained at 10 J. The number of impacts was set to 1, 5, 10, and 15, respectively, to analyze the variations in impact response, damage accumulation, and CAI strength of the laminates as the number of impacts increased. For the impacts at different locations, a two-impact sequence was adopted, with both impacts having an energy of 10 J. The impact spacing was set to 0 mm, 20 mm, 40 mm, and 80 mm. A spacing of 0 mm indicates that both impacts were located at the geometric center of the laminate, while spacings of 20 mm, 40 mm, and 80 mm correspond to the two impact points being offset by 10 mm, 20 mm, and 40 mm, respectively, to the left and right of the geometric center along the long edge direction of the laminate. These settings were used to analyze the effect of impact spacing on the impact response, damage propagation, and CAI strength of the laminates. Five replicate specimens were tested for each impact condition, including repeated impacts at the same location and double impacts at each impact spacing.

During low-velocity impact testing, the specimen edges were constrained by the fixture. Once the impact load was applied, the laminate exhibited both local contact deformation and global bending response, leading to transient coupled vibration among the impactor, specimen, and fixture. Because the clamped boundary was not perfectly rigid, the bending vibration of the specimen during impact was reflected in the contact force response. In addition, damage mechanisms such as matrix cracking and interlaminar delamination caused local stiffness changes, while the contact condition between the impactor and the specimen varied rapidly during loading and unloading. These factors collectively resulted in slight oscillations in the low-velocity impact response curves.

**Figure 5:**
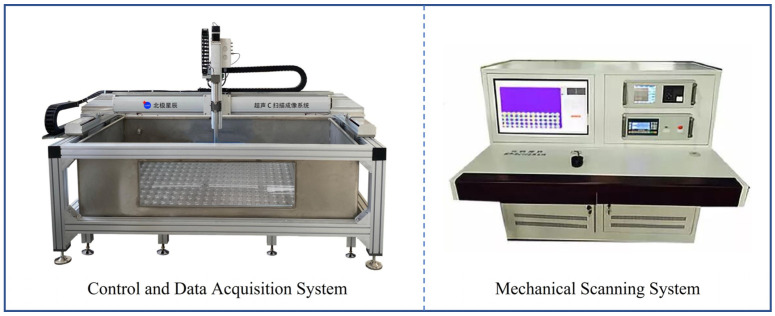
Ultrasonic C-scan system.

Composite laminates typically sustain internal damage that is difficult to identify visually during low-velocity impact. To obtain damage information of the specimens after impact, non-destructive testing was performed using a BSN-C1285 ultrasonic C-scan device (Beijing North Star Technology Co., Ltd., Beijing, China), as shown in Figure 5. This device consists of a computer-controlled data acquisition system and a mechanical scanning system, equipped with a 2.5P20 probe. It can automatically scan along the X and Y biaxial directions, with a probe movement speed of 300 mm/s and an inspection range of up to 1200 × 800 mm, enabling imaging of the internal damage morphology of the laminates. The C-scan damage area was calculated using ImageJ 1.54g. All C-scan images were exported with the same display range and resolution and then converted into 8-bit grayscale images. According to the grayscale histogram distribution in ImageJ, the damaged regions were mainly located within the low-grayscale range. Therefore, the damage identification threshold was set to 0–75. This threshold covered the clearly visible black and dark-blue damaged regions in the C-scan images while excluding red background textures, edge artifacts, and non-damage noise. After threshold segmentation, the damage area was calculated using the area measurement function in ImageJ.

**Figure 6:**
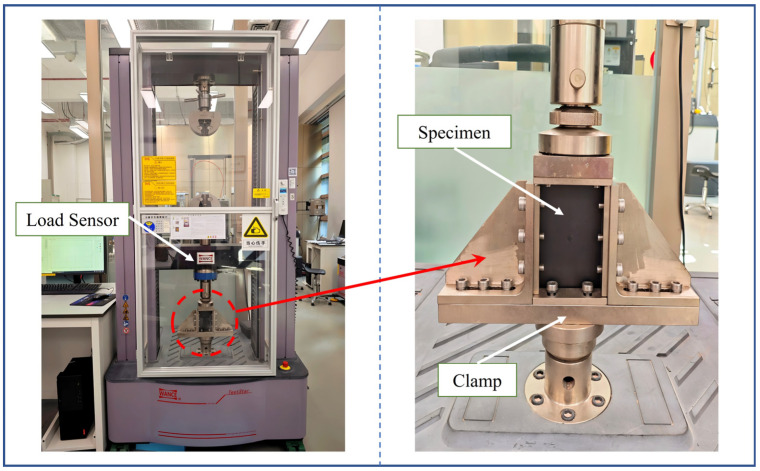
Compression-after-impact test setup.

Low-velocity impact can cause certain damage within composite components and reduce their subsequent load-bearing capacity. Compression-after-impact (CAI) tests are commonly employed to evaluate the damage tolerance of the components. The compression tests were conducted using a TSE105D universal electronic testing machine (Shenzhen Wance Testing Equipment Co., Ltd., Shenzhen, China), as shown in Figure 6. The machine has a maximum testing force of 100 kN, an accuracy class of 0.5, and a relative indication error of ±0.5% for the testing force. The accompanying TestPilot V3.0 software enables loading modes such as displacement control, force control, and strain control. During the tests, force and displacement data were acquired in real time by sensors. The tests were performed in accordance with ASTM D7137 [43]. The displacement loading rate during the formal loading stage was set to 1.25 mm/min. The CAI strength was calculated according to Equation (1):

(1) $$\sigma_{CAI}=\frac{F_{MAX}}{A}$$

where $\sigma_{CAI}$, $F_{MAX}$, and A represent the residual compressive strength, ultimate compressive load, and cross-sectional area of the specimen, with units in MPa, N, and mm^2^, respectively.

## 3. Experimental Results and Discussion

### 3.1. Investigation of Repeated Impacts

**Figure 7:**
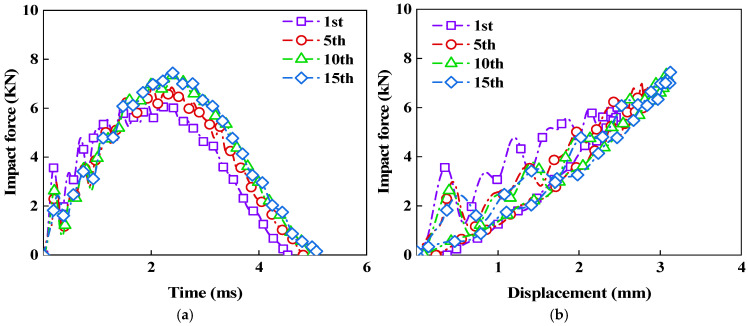
Impact response of CS laminates subjected to 1, 5, 10, and 15 repeated impacts at the same location: (**a**) Force–time curves; (**b**) Force–displacement curves.

As shown in Figure 7, after 1, 5, 10, and 15 consecutive impacts at the same location on the CS laminate, the overall shapes of the impact force–time and impact force–displacement curves are relatively similar, all exhibiting a typical loading-unloading process. There are noticeable fluctuations in the initial stage of the curves, which mainly occur at the beginning of the impact. These fluctuations are related to the local vibration, transient response, and damage initiation caused by the instantaneous contact of the impactor. However, both the time to reach the peak force and the total impact time gradually increase, indicating that the contact time between the impactor and the laminate continuously increases after repeated impacts. Meanwhile, as the number of impacts increases, the maximum central displacement continuously grows, causing the entire curve to shift to the right, indicating that the bending deformation of the laminate becomes more pronounced after repeated impacts.

Based on the initial linear region of the force–displacement curves, the stiffness values after 1, 5, 10, and 15 impacts were calculated as 2.50 kN/mm, 2.45 kN/mm, 2.39 kN/mm, and 2.39 kN/mm, respectively. During the first 10 impacts, the stiffness decreased from 2.50 kN/mm to 2.39 kN/mm, indicating that the accumulated damage altered the load transfer near the impact region and resulted in greater central displacement under the same load level. When the number of impacts increased from 10 to 15, the stiffness remained at 2.39 kN/mm, suggesting that the change in the initial loading response became less pronounced. At this stage, the main deformation region and local damage zone had already developed, and subsequent impacts primarily imposed repeated loading on the existing damaged area. The additional damage therefore had a limited effect on the bending load transfer path. In addition, repeated compression gradually compacted the contact region, leading to a more stable local contact response. Consequently, the stiffness did not continue to decrease.

It can also be observed that the area enclosed by the impact force–displacement curve continuously decreases as the number of impacts increases, indicating that the energy absorption capacity of the laminate gradually declines. These changes suggest that under consecutive impacts, internal damage within the laminate accumulates gradually, and the constraint effect in the local region is progressively weakened, making the laminate more susceptible to bending deformation, while the contact time also increases accordingly. On the other hand, repeated impacts tend to gradually compact the contact region, leading to a higher peak force exhibited by the CS bio-inspired spiral laminate during subsequent impacts.

Five parallel tests were conducted for each impact condition. Table 1 presents the standard deviations of the mechanical response parameters calculated from these five tests. The results show that the standard deviations of peak force, maximum central displacement, impact duration, absorbed energy, damage area, and CAI strength were generally low. Although the standard deviations of damage area and CAI strength were relatively larger, their scatter remained limited when considered in relation to the corresponding magnitudes of these parameters.

**Figure 8:**
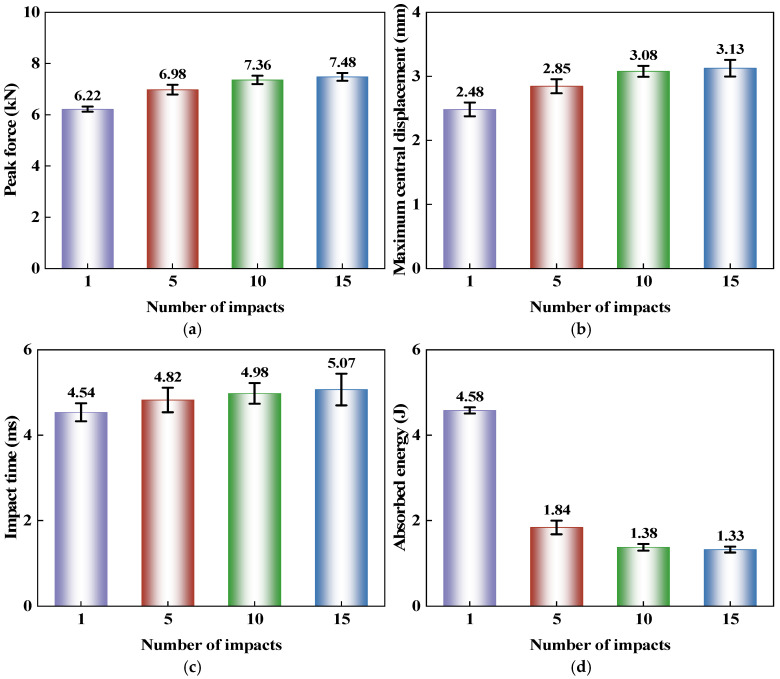
Force and energy response parameters of CS laminates subjected to 1, 5, 10, and 15 repeated impacts at the same location: (**a**) Peak force; (**b**) Maximum central displacement; (**c**) Impact time; (**d**) Absorbed energy.

To more intuitively compare the dynamic mechanical response characteristics of the CS laminate under different numbers of impacts, Figure 8 further presents the variations in peak force, maximum central displacement, impact time, and absorbed energy. As shown in Figure 8a, as the number of impacts increases from 1 to 15, the peak force of the CS laminate rises from 6.22 kN to 7.48 kN, exhibiting an overall upward trend. In terms of specific values, the peak force increases by 0.76 kN from 1 to 5 impacts, by 0.38 kN from 5 to 10 impacts, and by only 0.12 kN from 10 to 15 impacts, indicating that the peak force gradually stabilizes after consecutive impacts. This is because, after the initial impacts, the contact region gradually becomes compacted under repeated loading, leading to a change in the local contact state. As a result, higher loads are more easily achieved during subsequent impacts, causing the peak force to increase rapidly. With the accumulation of damage, matrix cracking and interlaminar damage continue to develop, affecting the load transfer within the laminate, and the rate of increase in peak force subsequently begins to diminish. The CS configuration also alters the propagation paths of intralaminar cracks and interlaminar damage, making it difficult for damage to rapidly concentrate locally. Therefore, a high level of contact load-bearing capacity can still be maintained after repeated impacts. As damage continues to propagate, this effect gradually weakens, and the change in peak load correspondingly slows down.

Figure 8b shows the variation in the maximum central displacement of the CS laminate with the number of impacts, and Figure 8c presents the corresponding trend of impact time. As the number of impacts at the same location increases, the maximum central displacement increases from 2.48 mm to 3.13 mm, exhibiting a continuous growth trend, with a more pronounced increase in the early stage and a gradually diminishing increase in the later stage. This trend indicates that the bending deformation of the laminate accumulates progressively after repeated impacts, making it easier to generate larger central displacements during subsequent impacts. In contrast, the impact time only increases from 4.54 ms to 5.07 ms, showing a relatively small overall change. Nevertheless, it still exhibits a slowly increasing trend, indicating that the influence of repeated impacts on the contact process is relatively limited, and the changes in structural response are primarily reflected in the development of deformation.

Figure 8d presents the experimental results of absorbed energy for the CS laminate under impact numbers of 1, 5, 10, and 15. As shown in the figure, the absorbed energy of the CS laminate continuously decreases with an increasing number of impacts at the same location, dropping from 4.58 J after a single impact to 1.33 J after 15 impacts. The most significant decrease occurs during the first few impacts. In terms of specific values, the absorbed energy decreases by 2.74 J from 1 to 5 impacts, while the reduction from 10 to 15 impacts is only 0.05 J. This result indicates that the first impact and the subsequent few impacts introduce new damage into the laminate, with a substantial portion of the impact energy being dissipated in damage formation and deformation development, leading to a rapid decline in absorbed energy. As the number of impacts continues to increase, the existing damage within the impacted region progressively accumulates, and subsequent loading primarily acts on the already damaged area. The energy dissipation associated with newly propagated damage is significantly reduced, causing the absorbed energy to gradually approach a stable level. Meanwhile, the spiral ply architecture makes the crack propagation paths more tortuous, facilitating damage deflection and dispersion during the initial impact stages, which allows more energy to be dissipated. Under consecutive impacts at the same location, the damage remains persistently concentrated near the original impacted region. The ability of the ply architecture to regulate subsequent damage propagation paths diminishes with an increasing number of impacts, and consequently, the structure’s capacity to dissipate impact energy also declines.

**Figure 9:**
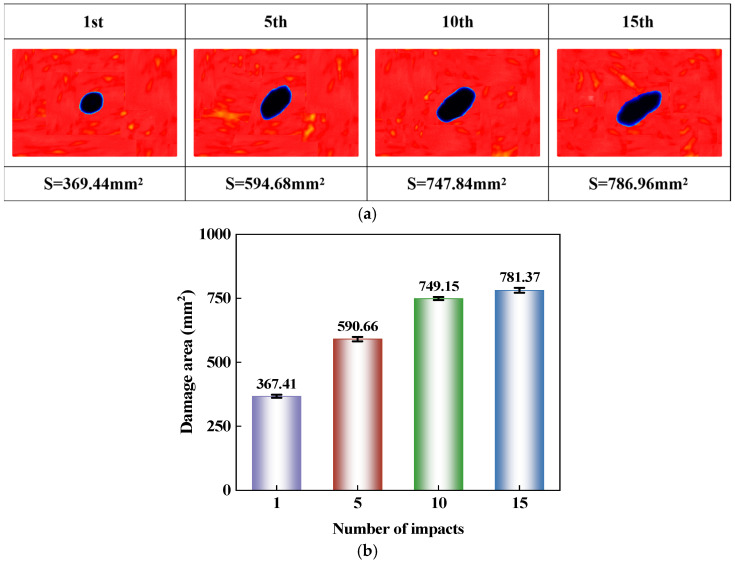
Ultrasonic C-scan images and damage area of CS laminates after 1, 5, 10, and 15 repeated impacts at the same location: (**a**) Ultrasonic C-scan images; (**b**) Damage area.

Based on the previous impact response results, the overall loading characteristics of the CS laminate under consecutive impacts at the same location have become relatively clear. However, the propagation extent and evolution direction of internal damage within the laminate require further analysis using ultrasonic C-scan results. Figure 9a presents the C-scan damage morphologies under different numbers of impacts, and Figure 9b shows the average damage area obtained from five replicate tests. As the number of impacts increases from 1 to 15, the damage area expands from 367.41 mm^2^ to 781.37 mm^2^. The propagation is more pronounced after the first few impacts, and the subsequent changes gradually slow down. In terms of damage morphology, the damage contours under the four impact numbers consistently maintain a relatively stable elliptical shape with an inclined orientation. The angle between the major axis direction and the horizontal line is approximately in the range of 35–45°.

During the first impact, the CS laminate remains close to its undamaged stiffness state, and fibers with different orientations in the cross-helical layup jointly carry the impact load. Consequently, deformation and interlaminar damage growth around the impact center are relatively uniform, resulting in an approximately circular damage area. As repeated impacts are applied at the same location, matrix cracking and delamination progressively accumulate near the impact center, leading to local stiffness degradation. Subsequent impacts therefore no longer act on an intact laminate, but promote damage growth from the pre-existing damaged region. In this process, damage associated with different fiber orientations in the cross-helical layup gradually becomes connected, and the local weakened region is elongated. As a result, the damage area grows more prominently in a specific direction, and its shape gradually evolves from nearly circular to elliptical. The inclination angle shows little variation across different impact numbers, indicating that although repeated impacts continuously drive the outward propagation of damage, the structural path governing damage propagation remains unchanged and is still controlled by the ply configuration. After the first impact on the laminate, delamination and matrix cracks have already formed within the plate. Subsequent impacts continue to act on the same impacted region, where the tips of existing damage and the damage boundaries become new initiation sites for further propagation. Therefore, the area growth is more rapid during the early stage. As the number of impacts further increases, the damage region within the impacted area has already become quite large, and subsequent loading primarily manifests as a slow outward expansion of the boundary. As a result, the damage area continues to increase, but the propagation rate begins to decline.

**Figure 10:**
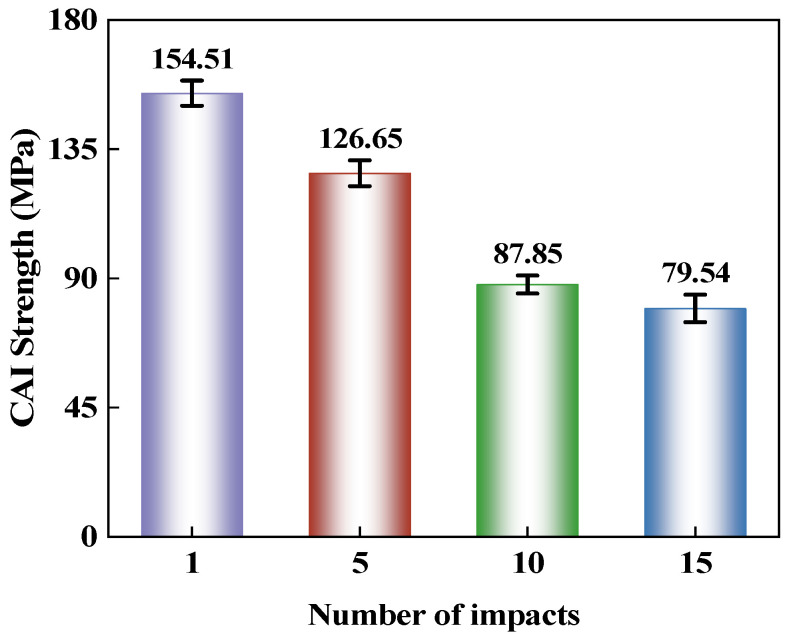
CAI strength of CS laminates after 1, 5, 10, and 15 repeated impacts at the same location.

The CAI strength reflects the residual capacity of the laminate to withstand compressive loads after low-velocity impact and serves as an important indicator for evaluating impact damage tolerance. The results are shown in Figure 10. As the number of impacts at the same location increases, the CAI strength of the CS laminate exhibits a continuous downward trend. This is primarily because the continuous accumulation and propagation of damage within the impacted region significantly increase the sensitivity of that region to compressive failure. With a further increase in the number of impacts, although subsequent impacts continue to drive the evolution of damage, their weakening effect on the residual compressive load-bearing capacity is no longer as pronounced as in the initial stage. Therefore, while the CAI strength continues to decrease, the rate of decline gradually slows down. For the CS spiral laminate, repeated impacts at the same location lead to continuous accumulation of local damage, which progressively evolves into the dominant region responsible for instability initiation and load-bearing capacity degradation during the compression stage, thereby playing an increasingly critical role in the overall failure process.

### 3.2. Influence of Impact Spacing

**Figure 11:**
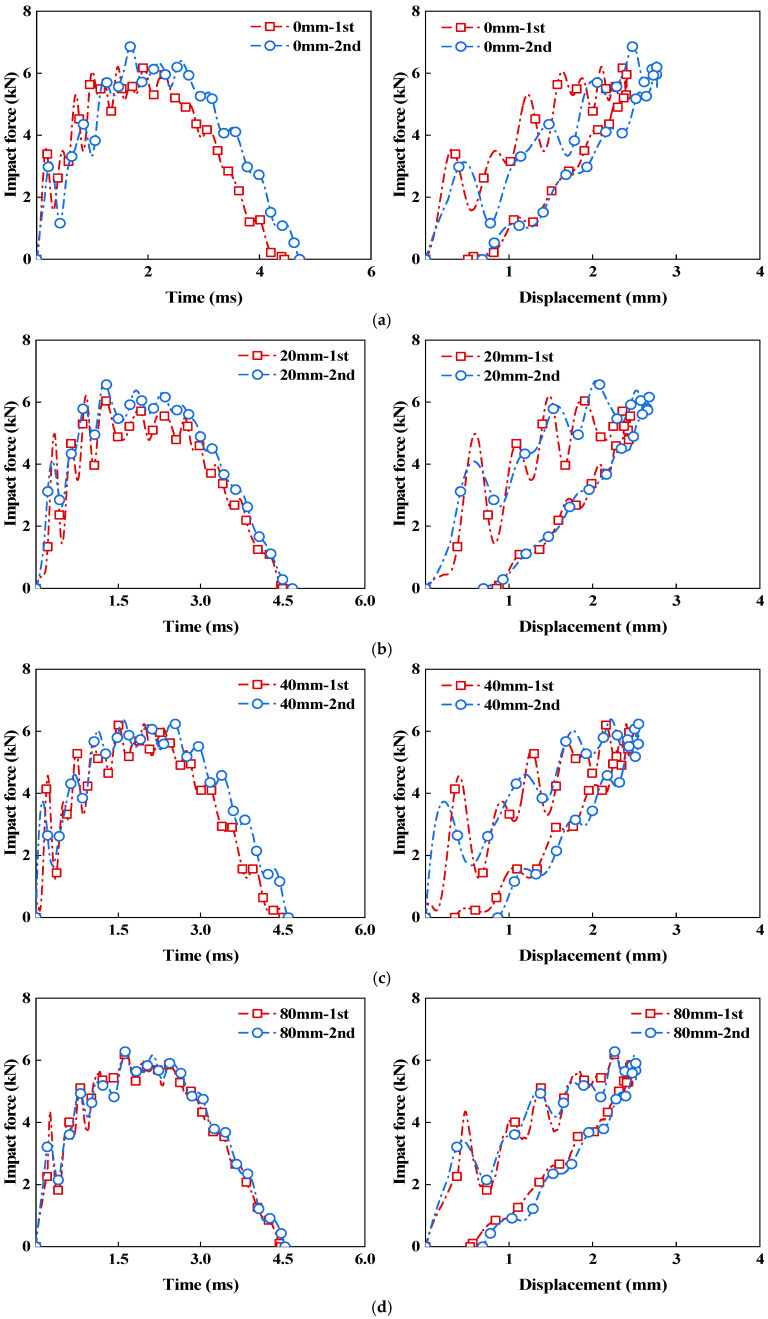
Force–time and force–displacement curves of CS laminates under double impacts with different impact spacings: (**a**) 0 mm; (**b**) 20 mm; (**c**) 40 mm; (**d**) 80 mm.

The impact force–time curves and impact force–displacement curves of the CS laminate subjected to two impacts, each with an energy of 10 J at different impact spacings, are shown in Figure 11, where Figure 11a–d correspond to impact spacings of 0 mm, 20 mm, 40 mm, and 80 mm, respectively. The purple curve represents the first impact, and the red curve represents the second impact. Overall, the response patterns of the two impacts remain consistent across different impact spacings; however, the relative positions of the two impact curves change significantly with varying impact spacing. When the impact spacing is 0 mm, the time to reach the peak load and the contact end time of the second impact are both later than those of the first impact, and the impact force–displacement curve also shifts entirely toward larger displacements, indicating that the damage caused by the first impact has significantly altered the contact state and deformation process during the second impact. After the impact spacing increases to 20 mm and 40 mm, the differences between the two impact curves still exist, but they show a clear trend of converging. At a spacing of 80 mm, the impact force–time curves of the two impacts are already quite close, and the differences in the impact force–displacement curves are further reduced, suggesting that the influence of the first impact on the response of the second impact has become relatively weak. Based on these results, it can be seen that when the impact spacing is small, the damage left by the first impact is more likely to affect the region of the second impact, and the local stiffness changes and damage accumulation have a more direct effect on the subsequent response. As the impact spacing increases, this mutual influence gradually weakens, and the differences in the responses between the two impacts continuously diminish.

**Figure 12:**
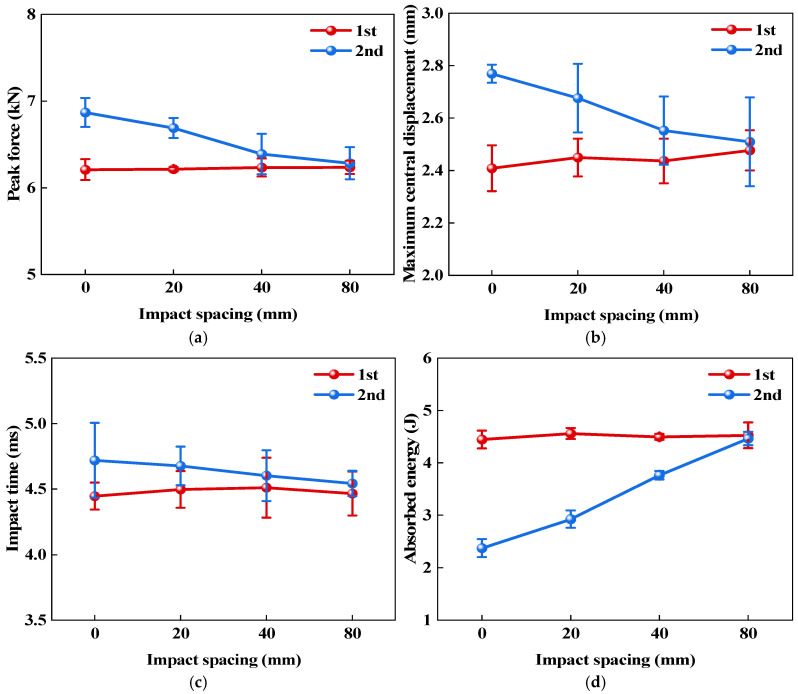
Impact response parameters of CS laminates under double impacts with different impact spacings: (**a**) Peak force; (**b**) Maximum central displacement; (**c**) Impact time; (**d**) Absorbed energy.

To further compare the effect of impact spacing on the double-impact response of the CS laminate, quantitative analyses were performed on peak force, maximum central displacement, impact time, and absorbed energy. As shown in Table 2, the standard deviations of the mechanical parameters of the CS laminates under different impact spacings were generally small, indicating low data scatter and good repeatability of the test results. From Table 3 and Figure 12, it can be seen that the variation in impact spacing has a minor effect on the first impact, with differences in parameters mainly reflected in the second impact. As the impact spacing increases, the differences in parameters between the two impacts generally decrease, indicating that the interference of local damage caused by the first impact on the response of the second impact has a limited spatial range. When the spacing is small, the second impact is more susceptible to the influence of prior damage and local stiffness changes; as the spacing increases, this interference gradually weakens, and the response of the second impact becomes closer to that of the first impact.

Figure 12a–d present the variations in peak force, maximum central displacement, impact time, and absorbed energy for the CS laminate under two impacts at different impact spacings. Specifically, (a), (b), (c), and (d) correspond to impact spacings of 0 mm, 20 mm, 40 mm, and 80 mm, respectively. Overall, the differences in the parameters of the first impact across the four spacings are very small, indicating good consistency among the specimens under the same 10 J impact condition, which indirectly reflects the stable manufacturing quality of the laminates. The changes brought about by impact spacing are mainly observed in the second impact. As the spacing increases from 0 mm to 80 mm, the peak force and maximum central displacement continuously decrease, while the absorbed energy steadily increases and gradually approaches that of the first impact. The impact duration shows little variation, exhibiting only a slowly shortening trend. These changes reflect that the damage coupling between the first and second impacts varies with the impact spacing. Under the condition of 0 mm spacing, the second impact directly acts within the damaged region formed by the first impact. The local stiffness has already decreased, and the existing damage has altered the subsequent contact state. As a result, larger deformation is more likely to occur, along with a higher peak load. After the first impact, a certain extent of internal damage has already formed in this region. The energy input during the second impact is primarily consumed in further deformation and propagation of the existing damaged area, leading to a relatively low level of absorbed energy. As the impact location gradually moves away from the main damage zone of the previous impact, the interference left by the previous impact continuously weakens. The deformation constraint and energy dissipation capacity in the local region begin to improve, resulting in a decrease in both peak load and maximum central displacement, while the absorbed energy increases correspondingly. Due to the cross-spiral structural characteristics of the CS laminate, the damage formed by the previous impact does not propagate straight through along a single interface. Under small spacing conditions, the influence of local damage coupling is more likely to be retained. As the impact spacing continues to increase, this local coupling effect gradually diminishes.

**Figure 13:**
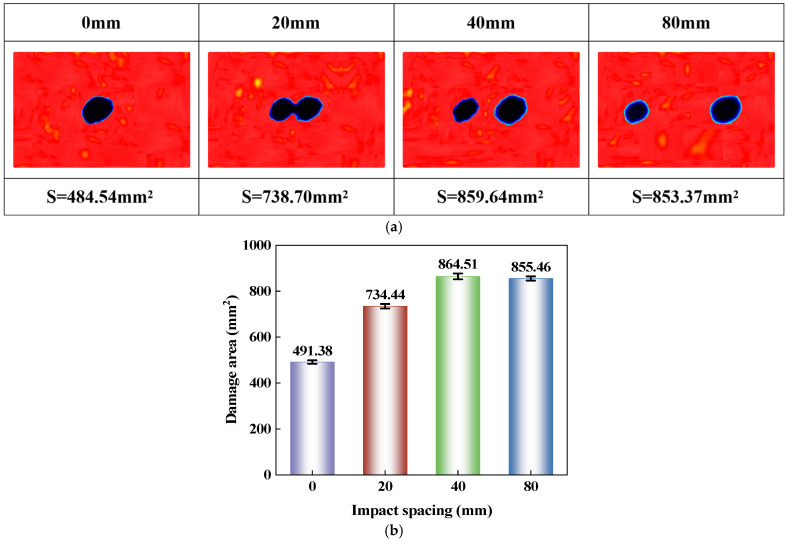
Ultrasonic C-scan results of CS laminates under double impacts with different impact spacings: (**a**) Damage morphology from ultrasonic C-scan; (**b**) Damage area.

The ultrasonic C-scan results of the CS laminate under different impact spacings are shown in Figure 13, where Figure 13a presents a comparison of damage morphologies, and Figure 13b shows the average damage area obtained from five replicate tests. As the impact spacing increases from 0 mm to 40 mm, the damage area expands from 491.38 mm^2^ to 864.51 mm^2^. When the spacing further increases to 80 mm, the damage area becomes 855.46 mm^2^, showing little change compared to that at 40 mm. As shown in Figure 13a, when the spacing is 0 mm, the two impacts act on the same location, and the C-scan image exhibits a single concentrated damage zone with an overall inclined elliptical contour that extends diagonally. In this case, the second impact primarily leads to continued propagation based on the existing damage, with most of the newly added damage overlapping with the already damaged area, resulting in a relatively small total damage area. When the impact spacing increases to 20 mm, the damage morphology begins to transition from a single zone to two distinct damage zones. Although the two damaged areas can be distinguished, there remains a clear connection between them, indicating that the delamination and cracks formed by the first and second impacts still exhibit strong spatial coupling. When the spacing increases to 40 mm, the two damage zones become further separated, while their contours still maintain similar inclined orientations, and the total damage area reaches its maximum. This is because the overlap between the damages caused by the two impacts has significantly weakened, while their mutual influence has not yet completely disappeared, making it easier to form a larger overall damaged region. At 80 mm spacing, the two damage zones are essentially independent in morphology, each propagating along its original direction. However, the total damage area does not continue to increase significantly; instead, it shows a slight decrease compared to that at 40 mm. This indicates that when the two impacts are further separated, the damage is primarily confined to the vicinity of each impacted region, and the overall area no longer expands with increasing spacing. In other words, the damage area at 80 mm corresponds to the total area of two separate damage zones, each propagating independently, rather than simply being double the area of the single damage zone at 0 mm spacing. This is because the damage range formed by each impact is still jointly constrained by local stiffness, ply architecture, and damage propagation pathways.

It should be noted that, although both impacts were performed at an energy of 10 J, the C-scan results for the 20 mm, 40 mm, and 80 mm spacing conditions show that the damage area associated with the second impact was larger than that caused by the first impact. This behavior is closely related to damage coupling during double-position impact loading. After the first impact, matrix cracking and interlaminar delamination had already developed within the laminate, reducing the local stiffness near the impacted region. During the subsequent impact, the pre-existing damage altered the load transfer path within the laminate, making the newly formed damage more likely to propagate along existing cracks and delamination interfaces. Therefore, even when the two impact locations were symmetric with respect to the geometric center, the second impact was still influenced by the damage state induced by the first impact, ultimately leading to a larger damage area in the C-scan image.

**Figure 14:**
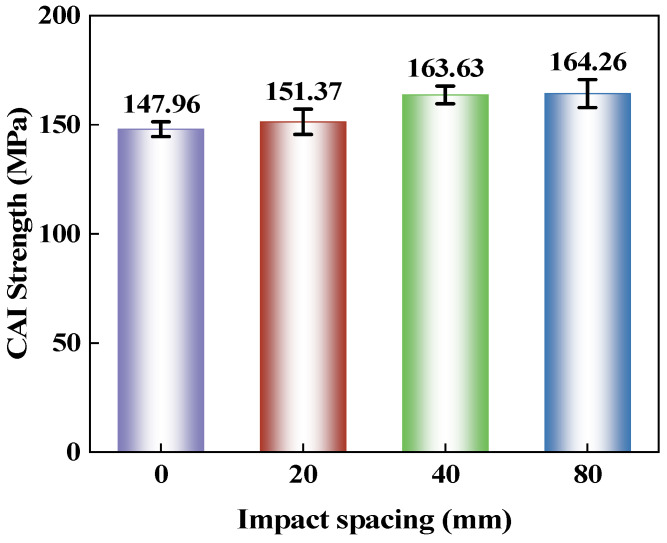
CAI strength of CS laminates under double impacts with different impact spacings.

Figure 14 shows that the impact spacing has a significant effect on the residual compressive load-bearing capacity of the CS laminate after double impacts. The CAI strength gradually increases as the impact spacing rises from 0 mm to 80 mm, improving from 147.96 MPa to 164.26 MPa. The increase from 0 mm to 20 mm is relatively small, while the enhancement from 20 mm to 40 mm is more pronounced. Between 40 mm and 80 mm, the CAI strength remains relatively stable. Combined with the previous results on damage morphology and damage area, under the condition of 0 mm spacing, the two impacts are concentrated at the same location, leading to continuous accumulation of local damage within the same region. This makes it easier for instability to initiate at that location under compressive loading, resulting in the lowest CAI strength. When the impact spacing increases to 20 mm and 40 mm, the damage caused by the first and second impacts gradually separates, and the damage overlap and local coupling are reduced. Consequently, the disturbance to compressive load transfer within the laminate is alleviated, and the residual compressive strength continuously improves. As the spacing further increases to 80 mm, the improvement in CAI strength becomes very limited. This is related to the fact that the damage from the two impacts is largely separated, and the local interaction is significantly weakened. In other words, at this spacing, the influence of the second impact on the compressive failure process depends more on the damaged regions themselves rather than being governed primarily by the superposition of the two damage zones. For the CS spiral laminate, the change in compressive performance after double impacts is closely related to whether significant overlap occurs between the damages induced by the first and second impacts.

## 4. Conclusions

This paper conducts an experimental study on the response characteristics of a bio-inspired cross-spiral (CS) laminate under multiple low-velocity impacts and compression after impact. The main conclusions are as follows:

(1) Consecutive impacts at the same location reduce the damage tolerance of the CS laminate. As the number of impacts increases from 1 to 15, the maximum central displacement increases from 2.48 mm to 3.13 mm, the absorbed energy decreases from 4.58 J to 1.33 J, and the damage area expands from 367.41 mm^2^ to 781.37 mm^2^. Repeated impacts promote the continuous accumulation of local damage, with the damage morphology gradually changing from a nearly circular shape to an inclined elliptical shape with a relatively stable orientation. During the CAI stage, the impacted region becomes more susceptible to local instability, resulting in a continuous reduction in CAI strength.
(2) Increasing the impact spacing weakens the damage coupling effect in the CS laminate under double-impact loading. As the spacing increases from 0 mm to 80 mm, the influence of the first impact on the second impact gradually diminishes, and the damage pattern changes from overlapping propagation to relatively independent growth. The damage area reaches 864.51 mm^2^ at a spacing of 40 mm and remains nearly unchanged at 855.46 mm^2^ when the spacing increases to 80 mm, indicating that further spacing increase does not lead to a notable expansion of the overall damaged region. The CAI strength increases from 147.96 MPa to 164.26 MPa, suggesting that reduced damage overlap lowers the susceptibility to local instability during compression and improves the residual load-bearing capacity.
(3) The spiral layup in the CS laminate alters the propagation paths of cracks and delaminations, causing damage to maintain a relatively stable directional propagation characteristic under multiple impacts, rather than rapidly developing along a single interface. However, when the local region is repeatedly impacted, damage continues to accumulate and induces local instability earlier during the subsequent compression stage, leading to a reduction in residual load-bearing capacity.
(4) This study has several limitations. The investigation was limited to CS laminates, without a direct comparison with conventional cross-ply or unidirectional laminates under identical material systems, specimen dimensions, and impact conditions. Therefore, the differences in impact resistance and damage tolerance among different layup configurations require further verification. In addition, the damage evolution under multiple low-velocity impacts was primarily analyzed based on experimental observations. Future work may incorporate finite element simulations to further examine damage initiation, propagation paths, and compression failure mechanisms.

## Figures and Tables

**Table 1:** Standard deviations of mechanical response parameters of CS laminates after 1, 5, 10 and 15 repeated low-velocity impacts (*n* = 5).

Code	Peak Force/kN	Maximum Central Displacement/mm	Impact Time/ms	Absorbed Energy/J	Damage Area/mm^2^	CAI Strength/MPa
1st	0.10	0.11	0.21	0.07	5.94	4.39
5th	0.19	0.11	0.29	0.16	8.71	4.51
10th	0.16	0.09	0.24	0.08	5.99	3.17
15th	0.15	0.13	0.37	0.07	9.82	4.76

**Table 2:** Standard deviations of mechanical response parameters of CS laminates under double impacts with different impact spacings (*n* = 5).

Code	Peak Force/kN	Maximum Central Displacement/mm	Impact Time/ms	Absorbed Energy/J	Damage Area/mm^2^	CAI Strength/MPa
0 mm-1st	0.12	0.07	0.10	0.17	7.64	3.41
0 mm-2nd	0.17	0.03	0.29	0.17		
20 mm-1st	0.02	0.08	0.14	0.10	10.02	5.81
20 mm-2nd	0.11	0.15	0.15	0.16		
40 mm-1st	0.10	0.07	0.23	0.06	12.84	4.08
40 mm-2nd	0.23	0.13	0.19	0.08		
80 mm-1st	0.08	0.08	0.17	0.25	9.32	6.41
80 mm-2nd	0.19	0.16	0.10	0.13		

**Table 3:** Impact response parameters of CS laminates under double impacts with different impact spacings.

Code	Peak Force/kN	Maximum Central Displacement/mm	Impact Time/ms	Absorbed Energy/J
0 mm-1st	6.21	2.41	4.45	4.49
0 mm-2nd	6.87	2.77	4.72	2.38
Difference	0.66	0.36	0.27	2.11
20 mm-1st	6.22	2.45	4.54	4.56
20 mm-2nd	6.69	2.68	4.68	2.93
Difference	0.47	0.23	0.14	1.63
40 mm-1st	6.23	2.44	4.51	4.5
40 mm-2nd	6.39	2.55	4.6	3.76
Difference	0.16	0.11	0.09	0.74
80 mm-1st	6.24	2.48	4.47	4.53
80 mm-2nd	6.28	2.51	4.54	4.46
Difference	0.04	0.03	0.07	0.07

## Data Availability

The raw/processed data required to reproduce these findings cannot be shared at this time as the data also forms part of an ongoing study.
